# P05.19. Outcomes in breast cancer treatment decision-making

**DOI:** 10.1186/1472-6882-12-S1-P379

**Published:** 2012-06-12

**Authors:** E McKenzie, A Mulkins, S Rose, M Verhoef

**Affiliations:** 1 University of Calgary, Calgary, Canada

## Purpose

This study compares decision-making (DM) and psychosocial characteristics of women with breast cancer who decline some, or all, conventional cancer treatment, and use CAM (cases), with those who accept all conventional cancer treatments and may or may not use CAM (controls).

## Methods

This case-control study involved telephone interviews at baseline, and completion of spirituality, self-efficacy, and control over treatment DM questionnaires at baseline, 6 months, and 1 year. Forty cases and 40 controls with stage 0-III breast cancer were matched by age, stage of illness, and province. Differences between pairs at each time point, adjusted for the time since diagnosis, were estimated using GEE linear regression models.

## Results

Seventy-five percent were 40-59 years old, and 80% diagnosed at stage I or II. Cases declined hormone therapy (67%), radiation (65%) and chemotherapy (63%), and used CAM therapies including diet changes (98%), mind-body practices (88%), and herbs (85%). Outcome measures are grouped as follows: (1) general health and wellbeing (Distress Thermometer (DT), Profile of Mood States (POMS); (2) impact of cancer on well-being (Functional Assessment of Cancer Therapy – Breast (FACT-B); and (3) measures of traits, such as personality and beliefs (Multidimensional Health Locus of Control, Spiritual Involvement and Beliefs Scale-Revised (SIBS-R), Control Preferences Scale (CPS), and Generalized Self-Efficacy. No significant differences in the DT (p>0.141) and POMS, except for the anger (p<0.001) and vigor (p=0.001) scales, were found. Cases exhibited higher anger and vigor scores. There were no differences between groups on the FACT-B subscales at any time point. Measures of traits demonstrated differences in spirituality (p<0.001) and control preferences in DM (p<0.001), but not self-efficacy (p=0.208).

## Conclusion

This study highlights the need for cancer specialists to assess individual needs, provide individualized care, and be open to a patient population with a wide range of beliefs.

